# Comparative transcriptome analysis reveals that tricarboxylic acid cycle-related genes are associated with maize CMS-C fertility restoration

**DOI:** 10.1186/s12870-018-1409-z

**Published:** 2018-09-12

**Authors:** Yongming Liu, Gui Wei, Yuanyan Xia, Xiaowei Liu, Jin Tang, Yanli Lu, Hai Lan, Suzhi Zhang, Chuan Li, Moju Cao

**Affiliations:** Key Laboratory of Biology and Genetic Improvement of Maize in Southwest Region of Ministry of Agriculture, Maize Research Institute, Sichuan Agricultural University, Chengdu, 611130 China

**Keywords:** Maize, Cytoplasmic male sterility, Fertility restoration, Oxoglutarate dehydrogenase, Isocitrate dehydrogenase, Transcriptome sequencing

## Abstract

**Background:**

C-type cytoplasmic male sterility (CMS-C) is one of the three major types of cytoplasmic male sterility (CMS) in maize. *Rf4* is a dominant restorer gene for CMS-C and has great value in hybrid maize breeding, but little information concerning its functional mechanism is known.

**Results:**

To reveal the functional mechanism of *Rf4*, we developed a pair of maize near-isogenic lines (NILs) for the *Rf4* locus, which included a NIL_*rf4* male-sterile line and a NIL_*Rf4* male fertility-restored line. Genetic analysis and molecular marker detection indicated that the male fertility of NIL_*Rf4* was controlled by *Rf4*. Whole-genome sequencing demonstrated genomic differences between the two NILs was clustered in the *Rf4* mapping region. Unmapped reads of NILs were further assembled to uncover *Rf4* candidates. RNA-Seq was then performed for the developing anthers of the NILs to identify critical genes and pathways associated with fertility restoration. A total of 7125 differentially expressed genes (DEGs) were identified. These DEGs were significantly enriched in 242 Gene Ontology (GO) categories, wherein 100 DEGs were involved in pollen tube development, pollen tube growth, pollen development, and gametophyte development. Homology analysis revealed 198 male fertility-related DEGs, and pathway enrichment analysis revealed that 58 DEGs were enriched in cell energy metabolism processes involved in glycolysis, the pentose phosphate pathway, and pyruvate metabolism. By querying the Plant Reactome Pathway database, we found that 14 of the DEGs were involved in the mitochondrial tricarboxylic acid (TCA) cycle and that most of them belonged to the isocitrate dehydrogenase (IDH) and oxoglutarate dehydrogenase (OGDH) enzyme complexes. Transcriptome sequencing and real-time quantitative PCR (qPCR) showed that all the above TCA cycle-related genes were up-regulated in NIL_*Rf4*. The results of our subsequent enzyme-linked immunosorbent assay (ELISA) experiments pointed out that the contents of both the IDH and OGDH enzymes accumulated more in the spikelets of NIL*_Rf4* than in those of NIL_*rf4.*

**Conclusion:**

The present research provides valuable genomic resources for deep insight into the molecular mechanism underlying CMS-C male fertility restoration. Importantly, our results indicated that genes involved in energy metabolism, especially some mitochondrial TCA cycle-related genes, were associated with maize CMS-C male fertility restoration.

**Electronic supplementary material:**

The online version of this article (10.1186/s12870-018-1409-z) contains supplementary material, which is available to authorized users.

## Background

Plant cytoplasmic male sterility (CMS) involves the shortage of functional pollen grains while female gametes are still normal. CMS is caused by a chimeric opening reading frame (ORF) resulting from spontaneous mitochondrial genome rearrangement [1]. Some nuclear genes that are referred to as restorer of fertility (*Rf*) genes can rescue CMS at different levels, such as the genomic, mRNA, protein, and metabolic levels [2–4]. The CMS/*Rf* system not only serves as an ideal model for studying communication between the nucleus and mitochondria but also represents a valuable tool for exploiting heterosis in hybrid seed production [2, 5]. At present, deep insight into plant CMS fertility restoration contributes to its wide utilization in hybrid seed production [6].

In maize, male-sterile cytoplasm can be divided into three categories according to the fertility restoration patterns displayed in the F_1_ hybrids: Texas (T), USDA (S) and Charrua (C) [7]. Among plants of these categories, C-type cytoplasmic male sterility (CMS-C) presents a stable male sterility and positively affects grain yield, contributing to its great application value in maize hybrid seed production [8, 9]. However, the fertility restoration mechanism of CMS-C appears to be highly complex. Previous studies have indicated that, with the exception of the two dominant *Rf4* and *Rf5* genes, quantitative trait loci (QTLs) are also involved in maize CMS-C fertility restoration [10–12]. These QTLs always partially restore CMS-C male fertility, which makes their use difficult. Moreover, *Rf*-*I* on chromosome 7 can inhibit the function of *Rf5* but not *Rf4* [13]. Furthermore, the genetic background of sterile lines might also affect the function of *Rf* genes in CMS-C, and the number of functional *Rf* gene might diverge when facing different male-sterile lines [14]. Overall, the complex mechanism of CMS-C fertility restoration brings about serious challenges to the application of CMS-C in hybrid seed production. *Rf4* can completely rescue most of the maize CMS-C lines and has great potential value in maize CMS-C hybrid popularization and utilization. Much work has been undertaken for *Rf4*, which was preliminarily mapped to the short arm of chromosome 8 [15–17]. *Rf4* was further mapped to a 1.5 cM interval (520 kb) that contained 12 candidate genes [18]. *Rf4* was ultimately narrowed down to a 12 kb region at the tip of chromosome 8; this region contains two nuclear-targeted genes: GRMZM2G021276 and GRMZM2G582028 [19]. However, additional experimental evidence is needed to confirm the *Rf4* candidate gene and to elucidate its functional mechanism.

Most restorer genes encode different pentatricopeptide repeat (PPR) proteins, enabling them both to be targeted to mitochondria where CMS genes and products are located and to bind to CMS transcripts [20, 21]. Surprisingly, no PPR-encoded genes were found in the final mapping region of *Rf4*, suggesting that *Rf4* might be a new type of restorer gene [18]. Elucidating the *Rf4* functional mechanism would be interesting, which may provide novel insights into plant CMS fertility restoration. However, the probability of *Rf4* encoding a PPR protein cannot be excluded because the reference genome of B73 is still incomplete and because B73 might even lack *Rf4*. In this study, comparative transcriptome sequencing was performed on *Rf4* near-isogenic lines (NILs) to reveal the functional mechanism of *Rf4*.

## Methods

### Plant materials

In this study, a nearly isogenic line at the *Rf4* locus (NIL_*Rf4*) was developed for comparative genome and transcriptome analyses. NIL_*Rf4* was developed from the maize inbred donor line A619 (*Rf4Rf4*) and the CMS-C male-sterile recipient line C698–3 (*rf4rf4*) (NIL_*rf4*). Moreover, the male-fertile plants (> 5 plants) were backcrossed with the plants of maintainer line 698–3 (*rf4rf4*) to produce the subsequent generation of the backcross population. Briefly, plants from the BC_4_ and BC_5_ populations were used for phenotype and genotype characterization, male-fertile plants from both the BC_5_ population and line C698–3 were used for comparative genome and transcriptome analysis, and male-fertile plants from the BC_6_ population were used for measurements of isocitrate dehydrogenase (IDH) and oxoglutarate dehydrogenase (OGDH) enzyme abundance. Specifically, fresh leaves from three NIL_*Rf4* or NIL_*rf4* individuals were separately mixed for genome sequencing. Moreover, spikelets at the pollen maturation stage with a length of 0.7–0.8 cm were collected from three individual fertile plants (NIL_*Rf4*_r1, NIL_*Rf4*_r2, and NIL_*Rf4*_r3) and three individual sterile plants (NIL_*rf4*_r1, NIL_*rf4*_r2, and NIL_*rf4*_r3) for transcriptome sequencing. The samples were frozen directly in liquid nitrogen and then stored at − 70 °C.

### Phenotype and genotype characterization

To characterize NIL_*Rf4*, the male fertility of each individual in backcross populations BC_4_ and BC_5_ was investigated. For male fertility, both anther exertion and pollen fertility were rated as we described previously [14]. Moreover, in that report, we developed a tightly linked *Rf4* marker (5′-CGCACCTAACCGTCTCC-3′, 5′-GCGCAAGTACGCCGTAC-3′). Here, the marker was used to confirm all plant genotypes in the BC_5_ backcross population. Genomic DNA from the fresh leaves of each plant was extracted using the modified cetyl-trimethylammonium bromide (CTAB) method [22]. PCR amplification was performed using Tsingke PCR Master Mix (Tsingke, China) in accordance with its specifications.

### Genome-Seq mapping, single nucleotide polymorphism (SNP) calling and annotation

Genomic DNA was isolated from the fresh leaves of NIL_*Rf4* (BC_5_) and NIL_*rf4* using a Plant Genomic DNA Kit (Tiangen, China). The concentration, purity, and integrity of the DNA samples were examined using 1% agarose gels, a NanoDrop 2000 Spectrophotometer and a Qubit fluorometer (Thermo Fisher Scientific, USA). Afterward, the two DNA composites were sent to Novogene Technology Co., Ltd. (Beijing, China) for library construction and sequencing. Paired-end sequencing libraries were prepared for genome sequencing on an Illumina HiSeq™ 2500 analyser (Illumina, USA). The length of the reads was 150 bp, and paired ends were obtained. Before mapping, we removed the adaptor fragments, low-quality sequences (≥ 50% of the bases with a quality score Q ≤ 5), and impurities (*N* > 10%). BWA 0.7.8 (parameters: mem -t 4 -k 32 -M -R) was used to map the clean reads against the B73 reference genome sequence (ftp://ftp.ensemblgenomes.org/pub/release-31/plants/fasta/zea_mays/dna/). SNP calling was performed with SAMtools 0.1.19 software [23]. Low-quality SNPs with a base quality value < 20 or with a read depth < 4 or > 1000 from the two composite sequences were excluded. The SNPs were categorized as exonic, intronic, intergenic, and splicing as well as within the untranslated region (UTR) based on their positions. Moreover, an average SNP index [24, 25] was calculated for both samples using a sliding window analysis with a 100 kb window size and a 10 kb increment.

### Identification and characterization of non-reference genes

Unmapped reads of NILs were used to assemble contigs by SOAPdenovo with default parameters. Novel gene models were predicted using Genewise and AUGUSTUS. Then, each novel gene putative function was annotated using the InterPro (http://www.ebi.ac.uk/interpro/), KEGG (https://www.kegg.jp/), Swissprot (https://www.uniprot.org/), TrEMBL (https://www.uniprot.org/), and GO (http://www.geneontology.org/) databases. HMMER 3.0 software with the Hidden Markov (HMM) profile of PPR domain (PF01535) was used to identify PPR genes among novel genes. TargetP (http://www.cbs.dtu.dk/services/TargetP/) was used to predict each novel gene subcellular location.

### RNA-Seq mapping

The total RNA of six samples, which included three NIL_*rf4* samples (NIL_*rf4*_r1, NIL_*rf4*_r2, and NIL_*rf4*_r3) and three NIL_*Rf4* (BC_5_) samples (NIL_*Rf4*_r1, NIL_*Rf4*_r2, and NIL_*Rf4*_r3), was respectively extracted using a Qiagen RNeasy Kit (Qiagen, USA). The concentration, purity, and integrity of the RNA samples were examined using 1% agarose gels, a NanoDrop 2000 Spectrophotometer (Thermo Fisher Scientific, USA), and an Agilent Bioanalyzer 2100 System (Agilent Technologies, USA). DNase I was used to digest the residual genomic DNA. The samples were subsequently sent to Chengdu Basebio Biotechnology Company (China) for library construction and transcriptome sequencing on an Illumina HiSeq™ 2500 platform (Illumina, USA). Raw reads (150 bp paired-end) were filtered in accordance with the same method used for Genome-Seq. Clean reads from the RNA-Seq were mapped via TopHat v2.0.9 to the same version of the B73 reference genome as that used for Genome-Seq. Only reads that uniquely mapped to the reference genome were used for further analysis.

### Identification of differentially expressed genes (DEGs) and functional analysis

Transcripts per million (TPM) was used to calculate the expression of each transcript [26]. Based on the normalized transcript expression, the *P*_values were corrected in accordance with the Benjamini-Hochberg method, and *Q*_value (corrected *P*_value) < 0.01 served as the threshold for screening the DEGs. All the DEGs were then subjected to Gene Ontology (GO) analysis (http://bioinfo.cau.edu.cn/agriGO/analysis.php) and Kyoto Encyclopedia of Genes and Genomes (KEGG) pathway analysis (http://www.genome.jp/kegg/) via hypergeometric tests. In the above enrichment analyses, the *P*_values were corrected with the default methods on the websites, and *Q*_value ≤0.05 was used as the threshold for selecting significantly enriched GO terms and KEGG pathways.

To identify the potential genes involved in altering male fertility, all the DEGs with an E_value <1e^− 30^ were queried in the Plant Male Reproduction Database (PMRD) (http://202.120.45.92/addb/), which contains 548 male fertility-related genes in *Arabidopsis*. The maize gene sequences were retrieved from MaizeGDB (ftp://ftp.ensemblgenomes.org/pub/plants/release-31/fasta/zea_mays). Afterward, all target gene expression data (fragments per kilobase of exon per million fragments mapped (FPKM)) in various tissues were obtained from qTeller (www.qteller.com). The FPKM values were log_2_ normalized (FPKM + 1), after which the Z-scores for each gene were calculated to assess their tissue expression specificity. Moreover, the corresponding information on PPR genes in maize was obtained from a recent report [27].

### Enzyme-linked immunosorbent assay (ELISA) detection of IDH and OGDH abundance in spikelets

A total of 100 mg of spikelets whose length ranged from 0.7–0.8 cm at the pollen maturation stage (the same stage as that used for transcriptome sequencing) of three individual sterile NIL_*rf4* plants and three individual fertile NIL_*Rf4* plants (BC_6_) was collected for IDH and OGDH content measurements. Each sample was fully pelletized in 900 μL of PBS (0.01 mol/L, pH = 7.2–7.4), chilled on ice and ultrasonicated for 30 min with 300 W of power to obtain mitochondrial IDH and OGDH. The homogenates were then centrifuged (5000 g, 10 min, 4°C), after which the supernatants were ultimately used for IDH and OGDH content measurements via ELISA kits (Nanjing SenBeiJia Biological Technological Co., Ltd., China) in accordance with the manufacturer’s protocol. Each sample was tested three times, and statistical differences were tested by independent Student’s *t*-tests.

### Real-time quantitative PCR (qPCR) analysis

To measure the expression levels of the tricarboxylic acid (TCA) cycle-related genes and validate the accuracy of the transcriptome sequencing, 12 and 9 genes were respectively selected to analyse their expression via qPCR (see primer information in Additional file 1). 18S RNA was used as an internal control. The RNA samples were prepared in the same manner as those used for RNA-Seq. One microgram of total RNA from each sample was used to synthesize cDNA via a PrimeScript™ RT Reagent Kit (TaKaRa, China) with RT-Primer Mix. qPCR was conducted on a CFX96™ Real Time system (Bio-Rad, USA) in conjunction with SYBR Green Real-time PCR Master Mix (TaKaRa, China). The comparative threshold cycle (CT) method (2^−ΔΔCt^) was then used to assess the relative expression level of each gene [28].

## Results

### Characterization of NIL_*Rf4*

Only two phenotypes, complete male fertility and complete male sterility, were observed in the BC_4_ and BC_5_ populations, and the male-fertile plants could form normal pollen and flowered normally (see Additional file 2). We observed a 1:1 ratio in terms of the number of plants that produced normal pollen and the number of plants that displayed aborted pollen in the BC_4_ and BC_5_ populations, indicating that male fertility was controlled by one single nuclear gene (see Additional file 3). In addition, we observed that the male fertility of the BC_5_ population co-segregated with the tightly linked the *Rf4* marker (see Additional file 4). The above results indicated that *Rf4* was successfully transferred to NIL_*Rf4*.

### Genomic profiles of NILs

**Fig. 1:**
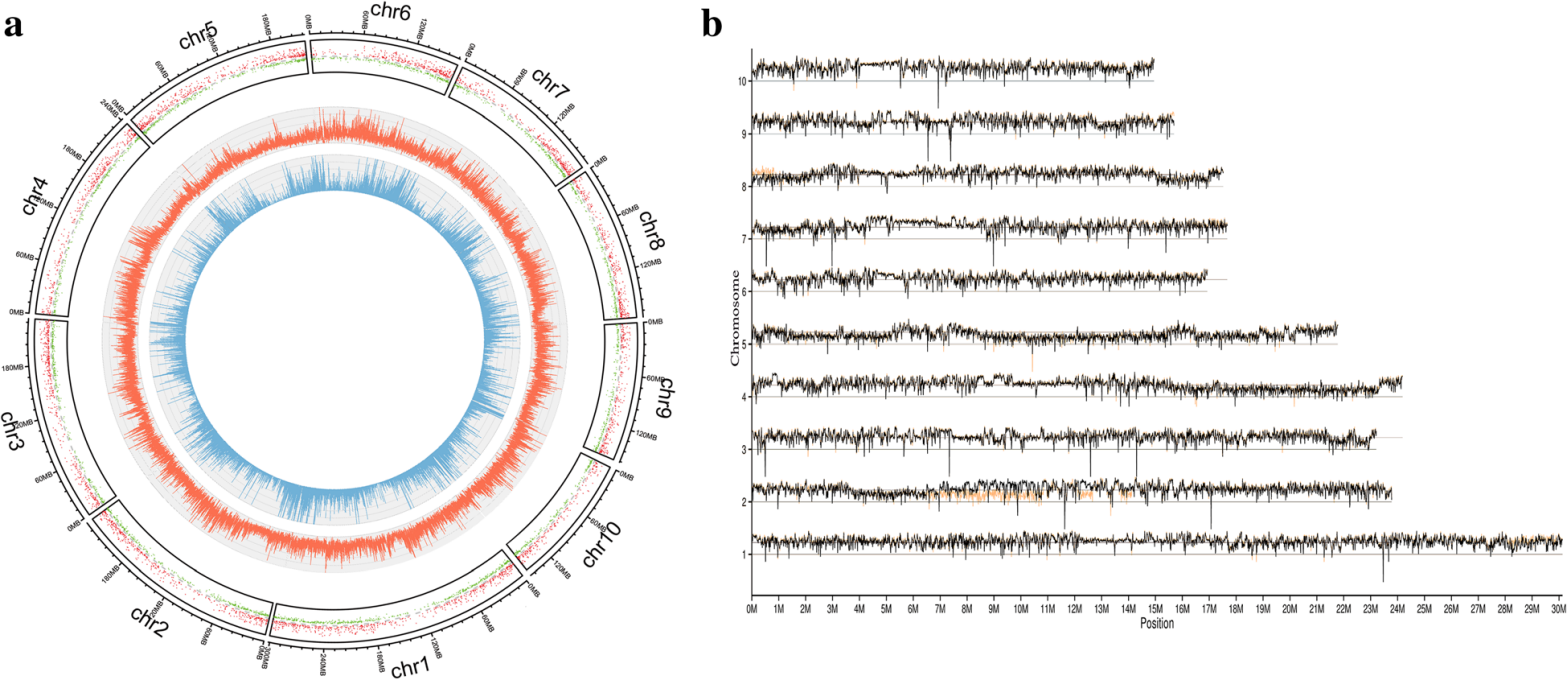
**a** Distribution of SNPs and DEGs between NIL_*Rf4* and NIL_*rf4*. From inner circle to the outer circle, the first and the second circles indicate the distribution of SNPs within exons and genome, respectively. In the third circle, the green dots represent up-regulated transcripts in NIL_*Rf4*, while the red dots represent down-regulated transcripts. **b** SNP index graphs of the NIL_*Rf4* (yellow lines) and NIL_*rf4* (black lines)

To reveal genomic variations between the NILs, polymorphic SNPs were detected using whole-genome sequencing. Total amounts of 98,331,340 and 83,494,528 short reads were obtained from NIL_*Rf4* (7.44× depth coverage) and NIL_*rf4* (6.48× depth coverage), respectively. These reads were then aligned to the B73 reference genome. A total of 1,216,260 SNPs between NIL_*Rf4* and NIL_*rf4* were identified throughout the whole genome, of which 26,383 SNPs were located in exons, 58,671 SNPs were located in introns, 169 SNPs were located at the variable splicing sites, 41,150 SNPs were located in UTRs, and 1,089,887 SNPs were located in intergenic regions (Fig. 1a). The above results showed that these SNPs were located mainly in noncoding regions. The genome sequencing results indicated that GRMZM2G122850 was the unique gene that harboured amino acid changes in the *Rf4* mapping region. GRMZM2G122850 encodes a selenium-binding protein that contains 390 amino acid residues; subcellular localization prediction via TargetP showed that this protein might be targeted to the mitochondria. Four SNPs were found to be located in its coding sequence and caused two missense mutations: R316H and D359N. These SNPs were confirmed via the dideoxy chain-termination method (see Additional file 5), and their potential effects need to be studied further.

Next, a SNP index was calculated for each identified SNP, and all SNP indexes of the two samples were plotted onto the whole maize genome (Fig. 1b). We found that the NILs shared similar SNP indexes across the majority of the maize genome. However, a series of biased SNP indexes were observed on the short arm of chromosome 8 from 0 to 1 Mb, which is consistent with the *Rf4* mapping region [19]. These results demonstrated that the genomic variations between NILs were located mainly near the *Rf4* locus.

### Putative functions of novel genes

As stated before, the reference genome of B73 may not be complete and it may even miss the sequence of *Rf4*. In this study, those unmapped reads were further assembled and annotated. We obtained 2890 putative genes in NILs (see Additional file 6) and most of their functions (> 90%) could be annotated (see Additional file 7). Among the novel genes, 645 genes could be targeted to the mitochondria and one PPR protein (Contig.4402.1) was found. These novel genes might help to uncover *Rf4* candidates in the future.

### Transcriptomic profiles of NILs

**Fig. 2:**
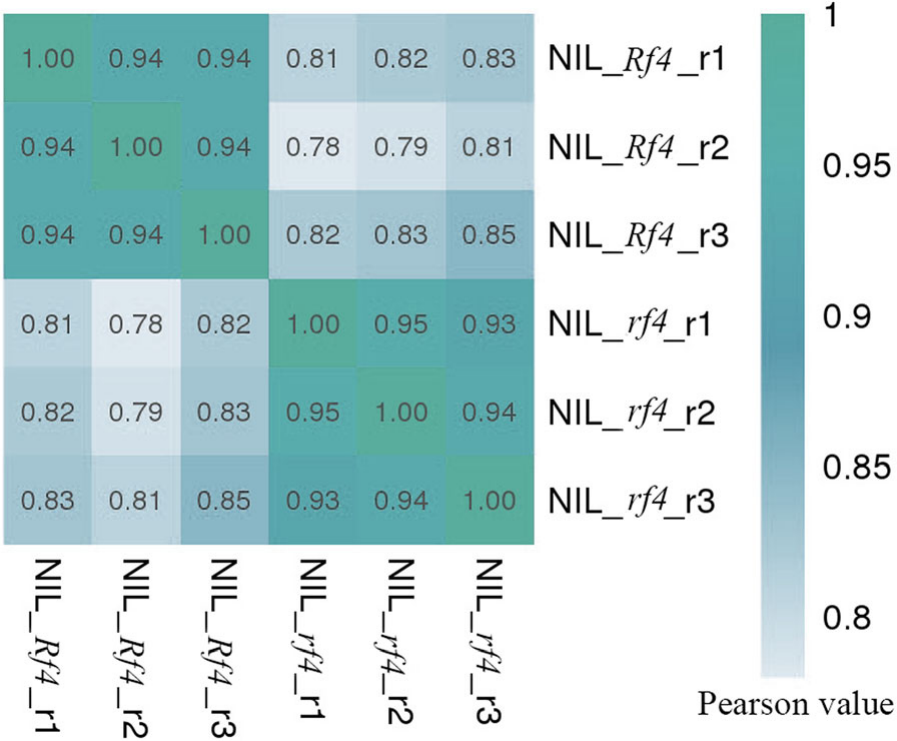
Pearson correlation coefficient analysis of the transcriptome data for three biological replicates

Six libraries, which consisted of three biological replicates per line, were prepared for RNA-Seq. A total of 265 million 150 bp paired-end reads were obtained for 6 samples; the reads for each sample ranged from 37 million to 54 million. The read length of all samples combined was > 37 Gb, representing an approximately 15-fold coverage of the maize genome. Approximately 71% of these short reads could be uniquely mapped to the reference genome (Table 1). Pearson correlation coefficient analysis revealed that the r^2^ value was greater than 0.93 for both groups and revealed both high correlations between and slight variability among biological replicates, showing the reliability of the transcriptome sequencing data (Fig. 2).

**Table 1:** Summary of RNA-Seq yields and alignments for six samples

Samples	Clean reads	Base (G)	Q20 (%)	Q30 (%)	GC (%)	Map rate (%)
NIL_*Rf4*_r1	39,893,186	5.98	97	92	57	73
NIL_*Rf4*_r2	44,696,350	6.70	97	93	59	72
NIL_*Rf4*_r3	37,224,984	5.58	97	92	59	73
NIL_*rf4*_r1	54,907,140	8.23	97	93	56	69
NIL_*rf4*_r2	43,082,408	6.46	97	93	57	68
NIL_*rf4*_r3	45,717,652	6.85	97	92	57	71

### Validation and functional characterization of DEGs

**Fig. 3:**
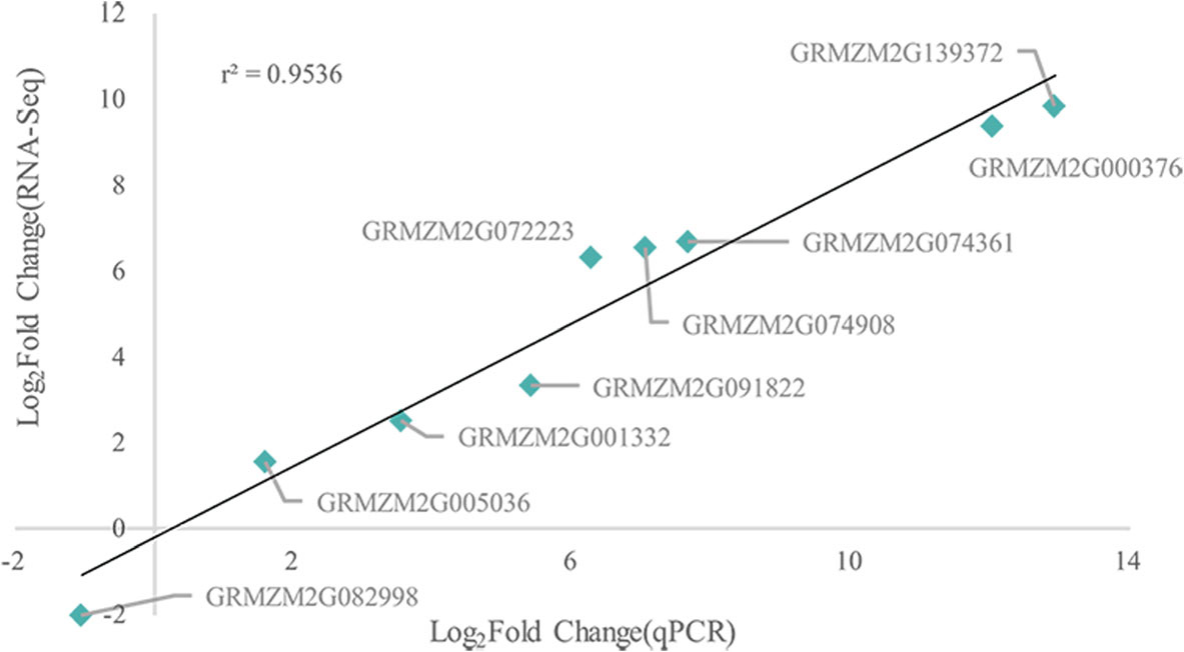
Correlation of gene expression levels estimated by RNA-Seq and qPCR. The x-axis and y-axis indicate relative expression values from qPCR and RNA-Seq, respectively. Both values are log_2_ normalized. The r value represents the Pearson correlation coefficient

To detect transcriptomic variation during male fertility conversion, the expression levels of all genes expressed in developing spikelets were compared between NIL_*Rf4* and NIL_*rf4* (Fig. 1a). In total, 7125 DEGs with 8125 transcripts were identified, the latter of which 91.22% (7740/8125) were up- or down-regulated by ≥2.0-fold. Among the 7125 DEGs, 3873 genes were up-regulated in NIL_*Rf4*, and 3252 genes were down-regulated. The abundance of DEGs suggested that changes in gene expression were active during the fertility restoration of CMS-C maize. By combining our Genome-Seq data, we identified a total of 1679 DEGs that had exonic SNPs throughout the whole genome (Fig. 1a and Additional file 8). Additional analyses of these DEGs might help to reveal the fertility restoration mechanism of *Rf4*. To verify the accuracy of the transcriptome changes revealed by RNA-Seq in this study, the transcript levels of nine randomly selected DEGs were subsequently examined via qPCR (Fig. 3). The expression patterns of these nine genes obtained by qPCR were highly consistent with the results of RNA-Seq (r^2^ = 0.9536), which confirmed the reliability of the transcriptome sequencing results.

**Fig. 4:**
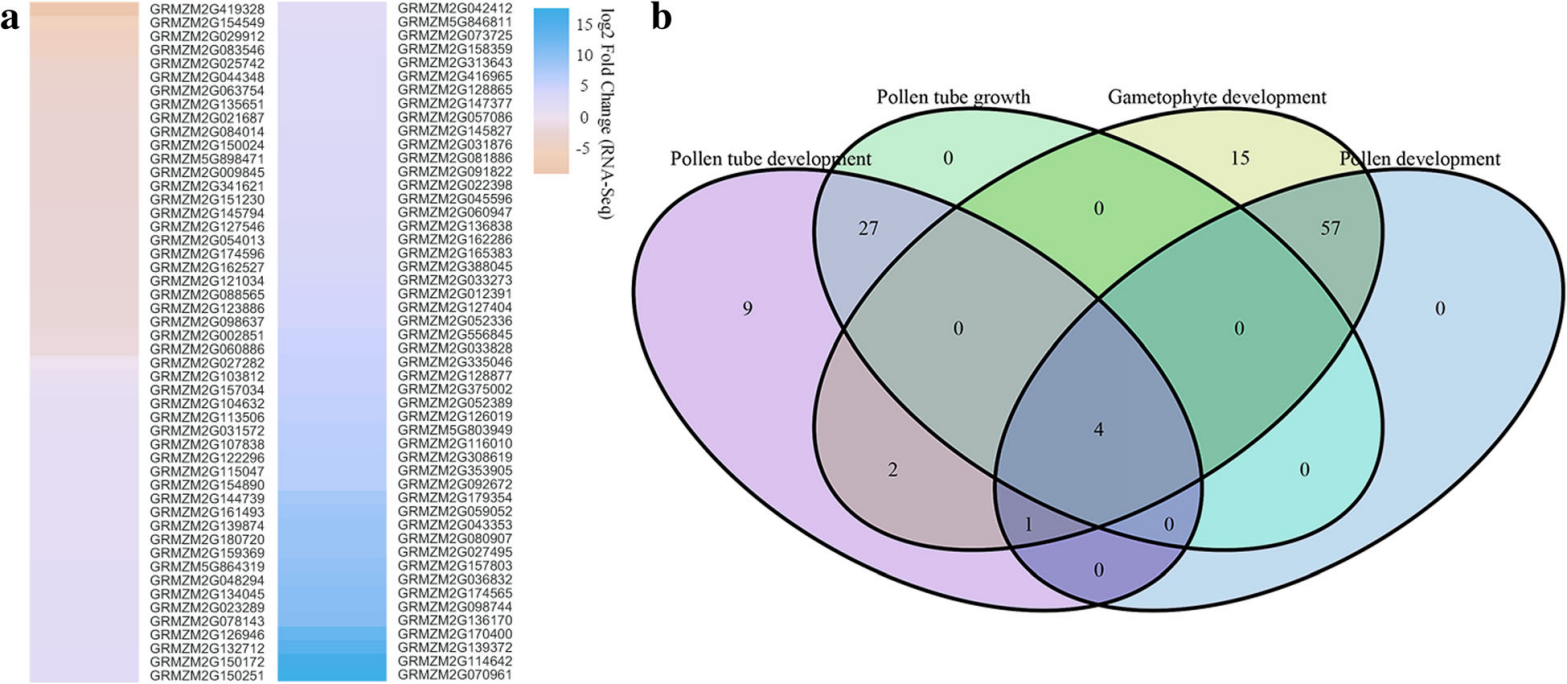
Expression **(a)** and Euler diagram **(b)** analyses of DEGs related to pollen development

To determine their putative function, the DEGs were subjected to GO analysis. A total of 5269 DEGs were annotated with 4810 GO terms. Within the biological process category, metabolic process, cellular process, and single-organism process were the top three abundant clusters. Under the cell component category, there were 451 enriched GO terms, and most of those genes were categorized as cell and cell part. With respect to the molecular function category, binding and catalytic activity were the most abundant subcategories. In addition, hypergeometric tests revealed 242 significantly enriched GO terms (see Additional file 9). Among the 242 GO terms, 4 terms were associated with 100 DEGs directly involved in pollen formation: pollen tube development (GO0048868), pollen tube growth (GO0009860), pollen development (GO0009855), and gametophyte development (GO0048229). RNA-Seq revealed that 74 of the 100 genes associated with the four abovementioned GO terms were up-regulated in the male-fertile NIL_*Rf4* plants (Fig. 4a), indicating that most of these genes play a positive regulatory role in maize pollen formation. Moreover, Euler diagram analysis revealed that most of these genes have various functions in pollen development (Fig. 4b). Importantly, four of them, GRMZM2G170400 (phosphoethanolamine N-methyltransferase 3), GRMZM2G060886 (S-adenosyl-L-methionine-dependent methyltransferase), GRMZM2G122296 (phosphoethanolamine N-methyltransferase 1-like), and GRMZM2G113506 (galacturonosyltransferase), are associated with all of the GO terms related to pollen development, implying their crucial effect on pollen development.

### Identification of male fertility-related DEGs

To identify key genes related to stamen development, the DEGs were aligned with 548 male *Arabidopsis* reproductive genes retrieved from the PMRD. A total of 198 genes were highly homologous to these *Arabidopsis* male reproductive genes; the cut-off E_value was <1e^− 30^ (see Additional file 10). Among these target genes, 63 were preferentially expressed in maize anthers and tassels (see Additional file 11), suggesting that they might play a critical role in maize male reproduction. Most of the Rf proteins are encoded by the PPR gene family. To reveal potential PPR genes involved in maize CMS-C fertility restoration, 56 differentially expressed PPR proteins were observed, among which 16 and 28 are located in the mitochondrion and chloroplast, respectively (see Additional file 12). However, only two differentially expressed PPR genes, ZmPPR432 (GRMZM5G851564) and ZmPPR443 (GRMZM2G050485), were found on chromosome 8, but both were far from the mapping region of *Rf4* and could not be the *Rf4* candidate gene.

### Identification of energy metabolism-related DEGs

Plant male fertility conversion is tightly associated with energy metabolism, and energy deficiency may cause male sterility [2, 29]. To gain a deep insight into the energy mechanism underlying CMS-C maize fertility restoration, 58 DEGs were found via KEGG analysis to participate in cell energy metabolism processes such as glycolysis, the pentose phosphate pathway, and pyruvate metabolism (Table 2). Among the 58 energy metabolism-related genes, 31 and 27 were up-regulated and down-regulated in NIL_*Rf4*, respectively. GRMZM2G154007, which encodes an alcohol dehydrogenase-like 2 protein, was the most up-regulated gene (fold change = 4096), whereas GRMZM2G345493, which encodes a fructose-bisphosphate aldolase 7 protein, was the most down-regulated gene (fold change = − 2048). Interestingly, two glycolysis-related genes (GRMZM5G824600 and GRMZM2G071630) were not expressed in NIL_*Rf4* or NIL_*rf4*, respectively. The different expression levels of the energy metabolism-related genes indicated the potential different energy levels between NIL_*rf4* and NIL_*Rf4* and that energy metabolism processes might play a key role in CMS-C fertility restoration.

**Table 2:** Annotations of energy metabolism-related DEGs

Gene ID	Log_2_ Fold Change	Direction^a^	Metabolism Process	Description
GRMZM2G345493	−11.00	down	Glycolysis/Pentose phosphate pathway	Fructose-bisphosphate aldolase 7 cytosolic
GRMZM5G836250	−9.81	down	Glycolysis/Pentose phosphate pathway	Fructose-1,6-bisphosphatase cytosolic
GRMZM2G134256	−7.14	down	Pentose phosphate pathway	Transaldolase 2
GRMZM2G046284	−6.94	down	Glycolysis/Pentose phosphate pathway	Fructose-bisphosphate aldolase
GRMZM2G046804	−6.85	down	Glycolysis	Glyceraldehyde-3-phosphate dehydrogenase, cytosolic 1
GRMZM2G001696	−6.84	down	Glycolysis/Pyruvate metabolism	Phosphoenolpyruvate carboxykinase1
GRMZM2G038791	−6.07	down	Pentose phosphate pathway	Ribose-phosphate pyrophosphokinase
GRMZM2G047592	−5.29	down	Glycolysis	Galactose mutarotase-like superfamily protein
GRMZM2G080375	−4.97	down	Glycolysis/Pentose phosphate pathway	ATP-dependent 6-phosphofructokinase 4 chloroplastic
GRMZM2G442658	−4.02	down	Glycolysis	Alcohol dehydrogenase 1
GRMZM2G155253	−3.39	down	Glycolysis/Pyruvate metabolism	Fructose-bisphosphate aldolase
GRMZM2G031107	−3.23	down	Pentose phosphate pathway	Glucose-6-phosphate 1-dehydrogenase
GRMZM2G083841	−3.18	down	Pyruvate metabolism	Phosphoenolpyruvate carboxylase 1
GRMZM5G852968	−2.91	down	Glycolysis	Triosephosphate isomerase
GRMZM2G306732	−2.78	down	Glycolysis/Pentose phosphate pathway	Fructose-1,6-bisphosphatase
GRMZM2G129513	−2.69	down	Pyruvate metabolism	Malate dehydrogenase 6
GRMZM2G026807	−2.39	down	Pentose phosphate pathway	Ribulose-phosphate 3-epimerase
GRMZM2G144730	−2.28	down	Glycolysis/Pyruvate metabolism	Pyruvate kinase
GRMZM2G127546	−2.09	down	Glycolysis/Pyruvate metabolism	Pyruvate dehydrogenase E1 component subunit beta-3 chloroplastic
GRMZM5G870932	−2.01	down	Glycolysis/Pyruvate metabolism	Phosphoenolpyruvate carboxykinase homologue 2
GRMZM2G415359	−1.96	down	Pyruvate metabolism	Malate dehydrogenase 5
GRMZM2G179521	−1.94	down	Pentose phosphate pathway	Glucose-6-phosphate 1-dehydrogenase
GRMZM5G879882	−1.92	down	Glycolysis/Pentose phosphate pathway	6-phosphofructokinase
GRMZM2G041356	−1.80	down	Glycolysis	Aldose 1-epimerase
GRMZM2G152975	−1.75	down	Glycolysis	Putative alcohol dehydrogenase superfamily protein
GRMZM2G033208	−1.55	down	Pentose phosphate pathway	Transketolase 1
GRMZM5G824600	No expression in NIL_*Rf4*	down	Glycolysis	Formaldehyde dehydrogenase homologue 1
GRMZM2G152981	0.40	up	Glycolysis	Putative alcohol dehydrogenase superfamily protein
GRMZM2G104632	1.50	up	Glycolysis	Glyceraldehyde-3-phosphate dehydrogenase, cytosolic
GRMZM2G057823	1.53	up	Glycolysis/Pentose phosphate pathway	Aldolase 1
GRMZM2G335657	1.56	up	Glycolysis/Pyruvate metabolism	Dihydrolipoyl dehydrogenase
GRMZM2G104081	1.64	up	Glycolysis	Hexokinase 1
GRMZM2G069542	1.85	up	Pyruvate metabolism	Phosphoenolpyruvate carboxylase 7
GRMZM2G466833	1.92	up	Pyruvate metabolism	Malate dehydrogenase3
GRMZM2G180625	1.93	up	Glycolysis	Cytosolic glyceroldehyde-3-phosphate dehydrogenase GAPC2
GRMZM2G023289	2.02	up	Glycolysis/Pentose phosphate pathway	Phosphoglucomutase 2
GRMZM2G043198	2.07	up	Glycolysis/Pentose phosphate pathway	Pyruvate dehydrogenase 2
GRMZM2G097226	2.15	up	Glycolysis/Pyruvate metabolism	Pyruvate dehydrogenase E1 beta subunit
GRMZM2G125233	2.28	up	Glycolysis	Galactose mutarotase-like superfamily protein
GRMZM2G440208	2.43	up	Pentose phosphate pathway	6-phosphogluconate dehydrogenase, decarboxylating
GRMZM2G098346	2.50	up	Glycolysis	Alcohol dehydrogenase 2
GRMZM2G066024	2.52	up	Glycolysis/Pentose phosphate pathway	Fructose-bisphosphate aldolase cytoplasmic isozyme
GRMZM2G139360	2.54	up	Glycolysis/Pentose phosphate pathway	ATP-dependent 6-phosphofructokinase 5 chloroplastic
GRMZM2G136918	2.73	up	Pentose phosphate pathway	Putative 6-phosphogluconolactonase 1
GRMZM2G058702	2.74	up	Glycolysis/Pyruvate metabolism	Dihydrolipoyllysine-residue acetyltransferase component 4 of pyruvate dehydrogenase complex chloroplastic
GRMZM2G162663	2.84	up	Glycolysis/Pyruvate metabolism	Acetyl-coenzyme A synthetase chloroplastic/glyoxysomal
GRMZM2G161245	2.96	up	Pyruvate metabolism	Malate dehydrogenase
GRMZM5G860137	3.01	up	Pyruvate metabolism	Acetyl-CoA acetyltransferase, cytosolic 2
GRMZM2G177947	3.12	up	Glycolysis/Pyruvate metabolism	Pyruvate kinase
GRMZM2G110714	3.18	up	Pyruvate metabolism	Phosphoenolpyruvate carboxylase 3
GRMZM2G074122	3.25	up	Pyruvate metabolism	Phosphoenolpyruvate carboxylase isoform 1
GRMZM2G322953	3.42	up	Glycolysis	Fructose-1,6-bisphosphatase, cytosolic
GRMZM2G003385	3.53	up	Glycolysis/Pentose phosphate pathway	2,3-bisphosphoglycerate-independent phosphoglycerate mutase 1
GRMZM5G886257	4.17	up	Pyruvate metabolism	NADP-dependent malic enzyme
GRMZM2G146206	4.19	up	Glycolysis	Triosephosphate isomerase, cytosolic
GRMZM2G003354	4.62	up	Glycolysis	Apospory-associated protein C
GRMZM2G154007	12.00	up	Glycolysis	Alcohol dehydrogenase-like 2
GRMZM2G071630	No expression in NIL_*rf4*	up	Glycolysis	Cytosolic glyceroldehyde-3-phosphate dehydrogenase GAPC3

^a^Direction means relative to the expression level in NIL_*Rf4*

### The abundance of IDH and OGDH in the mitochondrial TCA cycle were associated with fertility restoration

**Fig. 5:**
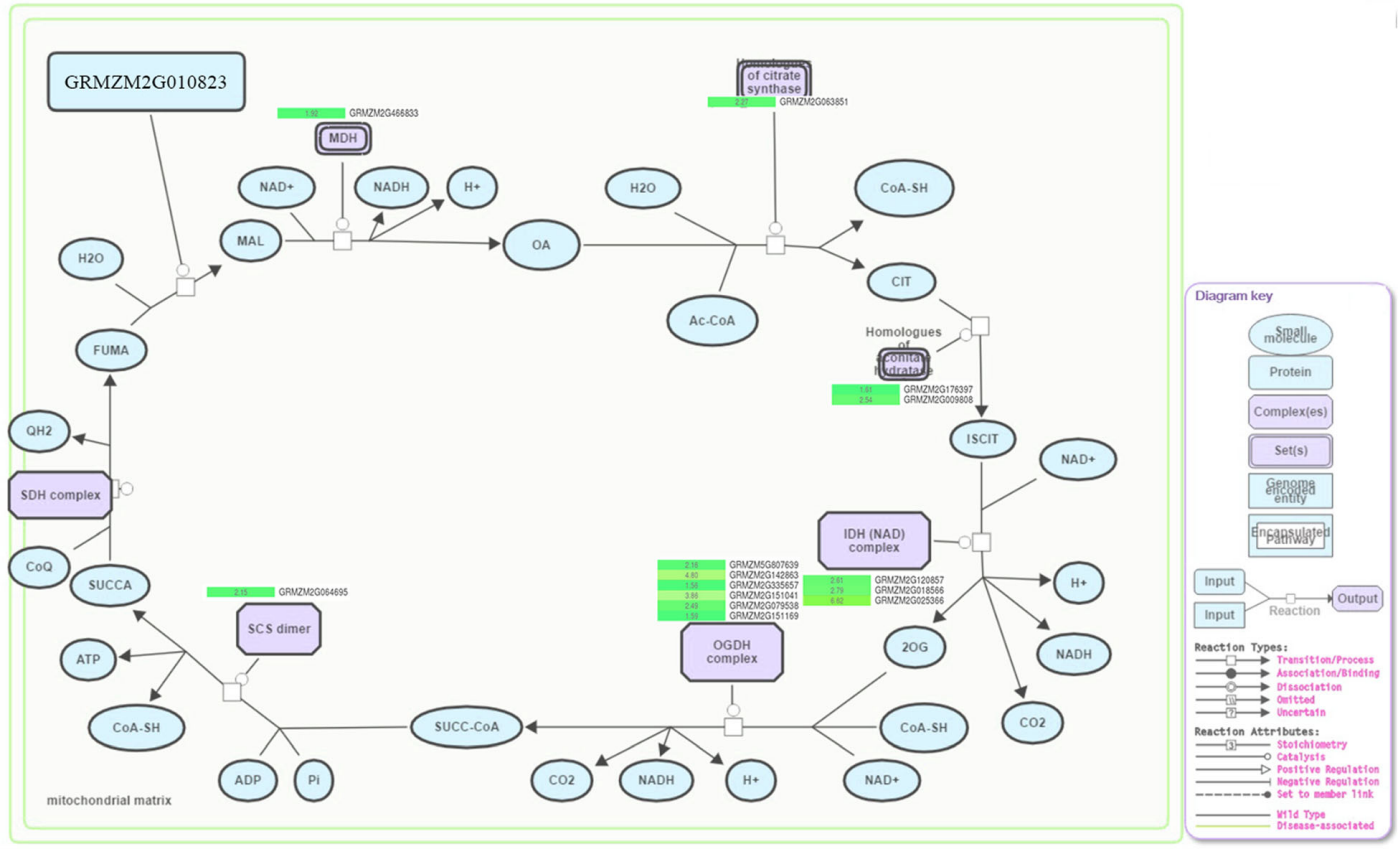
Identification of DEGs involved in the mitochondrial TCA cycle. The number indicates the gene expression fold changes that were log_2_ transformed in NIL_*Rf4* compared with NIL_*rf4*

**Fig. 6:**
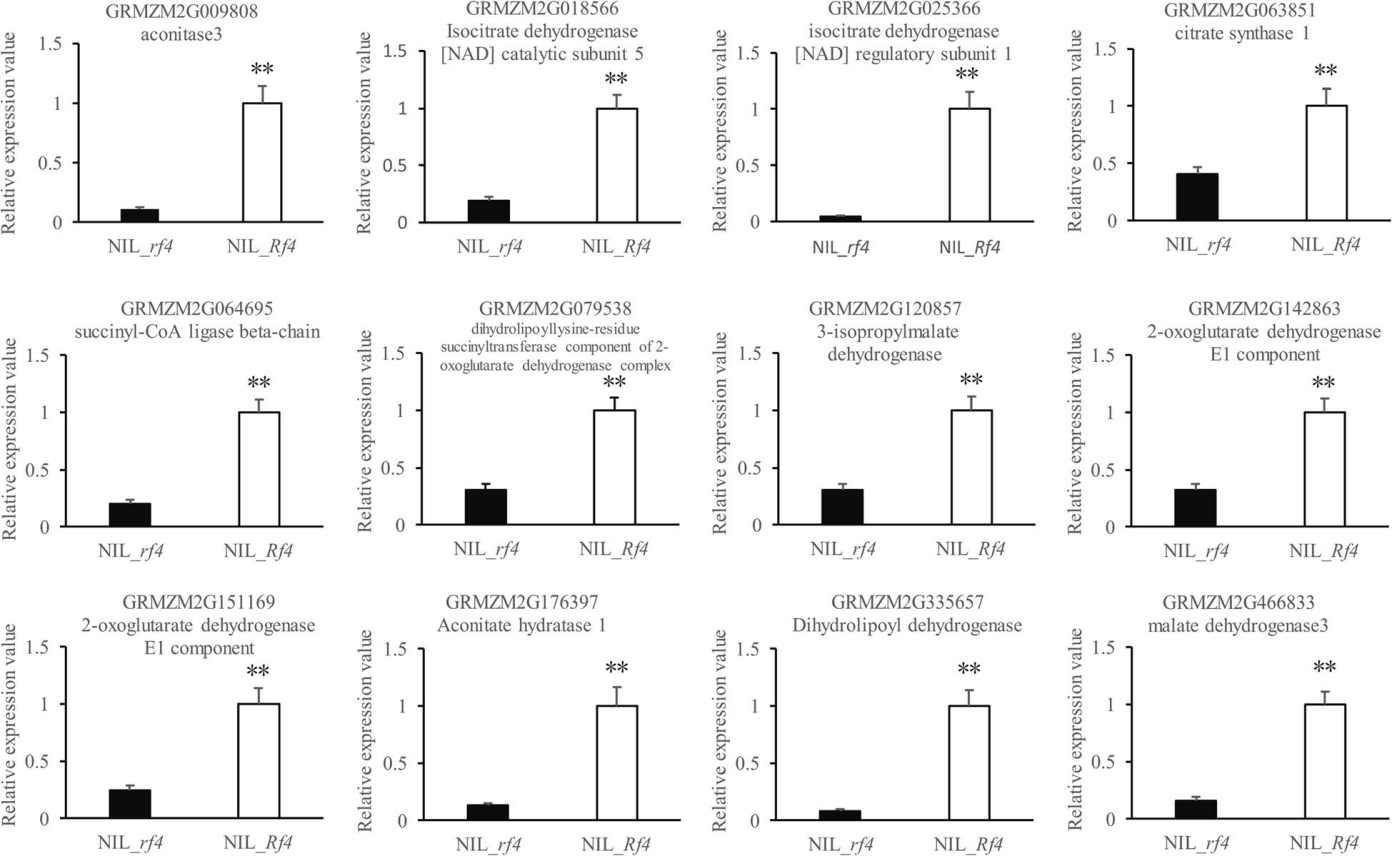
qPCR confirmation of genes associated with the mitochondrial TCA cycle. The data are given as means ± SEMs of three biological replicates. Independent Student’s *t*-tests were used to calculate the *P* values. **P < 0.01

**Fig. 7:**
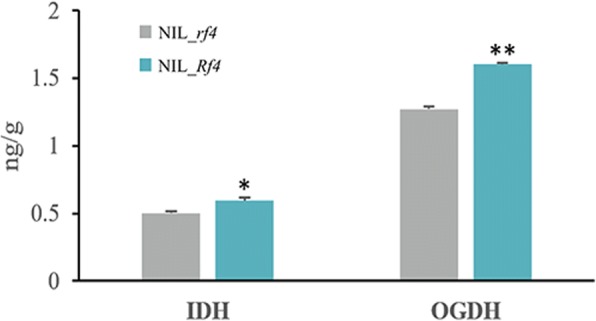
Comparison of IDH and OGDH enzyme levels between NIL_*Rf4* and NIL_*rf4*. The data are presented as the means ± SEMs of three biological replicates. Each replicate consisted of three technical replicates. The asterisks represent statistically significant differences between NIL_*Rf4* and NIL_*rf4* at *P* < 0.05 (*) or *P* < 0.01 (**) (Independent Student’s *t*-test)

Our KEGG analysis results above showed the probable different energy metabolism levels between NIL_*Rf4* and NIL_*rf4*. The mitochondrial TCA cycle plays a crucial role in energy metabolism [30]. As such, all DEGs were queried in the Plant Reactome Pathway database (http://plantreactome.oicr.on.ca/), and fourteen DEGs that participate in the mitochondrial TCA cycle were identified. Among these DEGs, GRMZM2G079538/GRMZM5G807639 and GRMZM2G142863/GRMZM2G151041 are duplicate genes. The RNA-Seq data demonstrated that these genes were all up-regulated in NIL_*Rf4* (Fig. 5), and the qPCR results confirmed the changes in their expression between NIL_*Rf4* and NIL_*rf4* (Fig. 6). Expression profile analysis revealed that these genes were expressed mainly in the anthers and tassels (see Additional file 13). We also used qPCR to explore the expression levels of these genes in developing anthers and leaves (see Additional file 13). For the maintainer line(HZS), the expression levels of these genes were similar between leaves and developing anthers; while for the CMS-C male-sterile line (CHZS), most of them were highly expressed in anthers during meiosis I. Compared with the maintainer line, all of these genes tended to show higher transcription levels at meiosis I in the male-sterile line. Three (GRMZM2G120857, GRMZM2G018566, GRMZM2G025366) and six (GRMZM2G807639, GRMZM2G151041, GRMZM2G142863, GRMZM2G335657, GRMZM2G079538, GRMZM2G151169) genes were found to participate in the IDH and OGDH complexes, respectively, both of which are key enzymes in the TCA cycle. Our additional ELISA experiments showed that the abundance of both IDH and OGDH was significantly higher in NIL_*Rf4* than in NIL_*rf4* (Fig. 7). The above results indicated that the genes related to the TCA cycle were associated with CMS-C male fertility.

## Discussion

As a dominant restorer gene for CMS-C, *Rf4* has great potential value in hybrid maize seed production. Understanding the function approach of *Rf4* will also benefit for revealing the abortive mechanism of maize CMS-C. Until now, the *Rf4* candidate gene had not been confirmed, and its functional mechanism was unknown. In this study, lines that were nearly isogenic at the *Rf4* locus were developed for comparative transcriptome analysis to uncover its function. The results of the genetic analysis and genotype characterization proved that the NIL_*Rf4* male fertility was restored just by *Rf4*. Genome-Seq was used to reveal the genomic variation in the NILs, and the results confirmed that most of the SNPs were crowded near the *Rf4* location. However, many SNPs were also gathered near the middle of chromosome 2. Various genomic differences clustering in the non-object area of NILs have also been reported in other studies [31]. In fact, NILs always present extensive residual heterozygosity because of the complexity of genomic composition and recombination [32, 33]. Moreover, previous studies in which SNP chips were used have generally reported the presence of SNPs between NILs [34–36]. Compared to SNP chips, next-generation sequencing has a broader and more sensitive detection ability, which might also explain why we detected more SNPs than did previous researchers.

Insufficient energy supplies can impair male gamete development but not vegetative organs, ultimately causing male sterility [2]. Previous studies have also shown that a close relationship exists between pollen dysfunction and energy metabolism in CMS-C. Three ATP-synthesizing chimeric ORFs, i.e., *atp9-c*, *cox2-c*, and *atp6-c*, were first identified in CMS-C plants [37]. Nevertheless, Meyer did not observe any chimeric proteins but did report that the expression of several ATP synthase complex proteins decreased within CMS-C tassels [38]. It can be inferred that pollen abortion in CMS-C plants is caused by inadequate ATP production because of the low atp9-c translation efficiency. Moreover, our group analysed the differences between the male-sterile line (C48–2) and its maintainer line N48–2, and we found that some DEGs and differentially expressed proteins were related to energy metabolism [39, 40]. At the metabolic level, compared to the anthers of the maintainer line, the anthers of the CMS-C line always present lower levels of respiratory intensity, cyanide-resistant respiration, cytochrome oxidase activity and ATP content during microspore development [41, 42]. In the present study, elevated IDH and OGDH enzyme levels were detected in the male fertility-restored line. Both IDH and OGDH are NADH-synthesizing enzymes and play a key role in the mitochondrial TCA cycle, which determines the energy supply in cells [29, 30]. Down-regulation of the mitochondrial citrate synthase enzyme ultimately causes male sterility [43]. Our results provided new evidence for the association between the mitochondrial TCA cycle and plant male fertility. Interestingly, when compared with the CMS-C male-sterile line, these TCA cycle-related genes were up-regulated in anthers of NIL_*Rf4* while down-regulated in maintainer line anthers. These results revealed the probable existence of different energy metabolism-related genes expression levels between the maintainer line and NIL_*Rf4* although they both were male fertile. This hypothesis needs to be further verified because the difference might also be caused by different pollen developmental stages in comparison.

With the exception of those in maize, many studies about CMS in other species show that, compared with normal lines, CMS lines always appear to have lower energy metabolism levels [44–46]. Mitochondria play an essential role in supplying cellular energy. Most mitochondrial proteins are encoded by nuclear genes; these proteins are synthesized in the cytoplasm and then imported into the mitochondria [47]. Recently, increasing amounts of attention have been paid to the connection between these nuclear-encoded mitochondrial proteins and CMS fertility restoration. *Rf2* encodes a mitochondrial aldehyde dehydrogenase (ALDH) enzyme, and its expression can rescue maize T-type cytoplasmic male sterility (CMS-T) [48, 49]. Both GRP162 and RFC3 proteins accumulate within mitochondria and can directly interact with *Rf5* to rescue HongLian cytoplasmic male sterility (HL-CMS) in rice [50, 51]. Hexokinase 6 is a mitochondrial-localized protein whose interaction with *Rf6* is necessary for HL-CMS fertility restoration [52]. This report provided direct evidence that energy metabolism-related genes directly contribute to plant CMS fertility restoration. In our experiments, 14 up-regulated genes involved in the TCA cycle were identified; however, their detailed function in restoring the fertility of maize CMS-C requires further study.

## Conclusions

Lines that were nearly isogenic at the *Rf4* locus were developed and characterized via genetic analysis, molecular marker detection and whole-genome sequencing. Comparative transcriptome analysis between male-fertile NIL_*Rf4* plants and male-sterile NIL_*rf4* plants revealed several crucial genes and metabolic pathways during CMS-C male fertility restoration. Interestingly, all of the DEGs involved in the mitochondrial TCA cycle were up-regulated in the male-fertile NIL_*Rf4* plants, and most of those DEGs tended to be involved in the IDH and OGDH complexes. Our ELISA results demonstrated that the abundance of both the IDH and OGDH enzymes was higher in NIL_*Rf4* than in NIL_*rf4*, indicating that the IDH and OGDH enzymes might be related to maize CMS-C fertility restoration. These results expand the current understanding of maize CMS-C male fertility restoration.

## Additional files

Additional file 1: List of primers used for qPCR in this study. 18S RNA was used as an internal control. (XLSX 12 kb)

Additional file 2: Comparisons of male-fertile tassels **(a)**, anthers **(b)**, and pollen **(c)** between NIL_*Rf4* and NIL_*rf4*. In Fig. **(c)**, the pollen grains were stained with 1% (*w*/*v*) KI-I_2_. (TIF 787 kb)

Additional file 3: Fertility segregations of the BC_4_ and BC_5_ populations. A chi-squared test was used to evaluate the fertility segregation ratios. (XLSX 11 kb)

Additional file 4: Genotype analysis of the BC_5_ population via the dominant *Rf4* tightly linked marker (5′-CGCACCTAACCGTCTCC-3′, 5′-GCGCAAGTACGCCGTAC-3′). F, fertile individuals; S, sterile individuals. (TIF 129 kb)

Additional file 5: Validation of SNPs within the coding sequence of GRMZM2G122850. (TIF 191 kb)

Additional file 6: Coding sequences of novel genes. (TXT 1323 kb)

Additional file 7: Function annotations of novel genes. (XLSX 315 kb)

Additional file 8: All DEGs with exonic SNPs. (XLSX 504 kb)

Additional file 9: Two hundred forty-two significantly enriched GO terms obtained by Fisher’s test (*Q* ≤ 0.05). (XLSX 20 kb)

Additional file 10: DEGs homologous to *Arabidopsis* male sterility (MS)/reproduction (MR) genes. (XLSX 35 kb)

Additional file 11: Expression patterns of male reproduction-related DEGs. Their expression values (FPKM) were retrieved from the qTeller website (www.qteller.com), and the FPKM values were log_2_ transformed by (FPKM + 1); a Z-score was then calculated for each gene. (TIF 298 kb)

Additional file 12: Identification of PPR genes among DEGs. “Direction” means relative to the expression level in NIL_*Rf4*. (XLSX 13 kb)

Additional file 13: **(a)**Tissue expression profile of 14 TCA cycle-related DEGs. The expression data (FPKM values) were obtained from qTeller (www.qteller.com). The FPKM values were log_2_ transformed (FPKM + 1), and a Z-score was calculated for each gene. **(b)** Comparison of TCA-related DEGs expression levels between the male-sterile line CHZS and its maintainer line HZS. A1, A2, and A3 denote developing anthers with the length of 1.5~ 2.0 mm (meiosis I), 2.0~ 2.5 mm (meiosis II) and 2.5~ 3.0 mm (uninucleate microspore) respectively. The data are given as means ± SEMs of at least three biological replicates. The relationship between maize pollen development stage and anther lenght can be found in another report [53]. (TIF 466 kb)

## Abbreviations

ALDH: aldehyde dehydrogenase
CMS: cytoplasmic male sterility
CMS-C: C-type cytoplasmic male sterility
CMS-T: T-type cytoplasmic male sterility
DEGs: differentially expressed genes
ELISA: enzyme-linked immunosorbent assay
FPKM: fragments per kilobase of exon per million fragments mapped
GO: Gene Ontology
HL-CMS: HongLian cytoplasmic male sterility
HMM: Hidden Markov Motif
IDH: isocitrate dehydrogenase
KEGG: Kyoto Encyclopedia of Genes and Genomes
NIL: near-isogenic line
OGDH: oxoglutarate dehydrogenase
ORF: opening reading frame
PMRD: Plant Male Reproduction Database
PPR: pentatricopeptide repeat
qPCR: real-time quantitative PCR
QTL: quantitative trait locus
Rf: restorer of fertility
SNP: Single nucleotide polymorphism
TCA: tricarboxylic acid
TPM: transcripts per million
UTR: untranslated region
