# Supplementary figures and images for: Comparative transcriptome analysis reveals that tricarboxylic acid cycle-related genes are associated with maize CMS-C fertility restoration
Source: BMC Plant Biol. 2018 Sep 12;18:190. doi: 10.1186/s12870-018-1409-z (PMC6136215; doi:10.1186/s12870-018-1409-z)

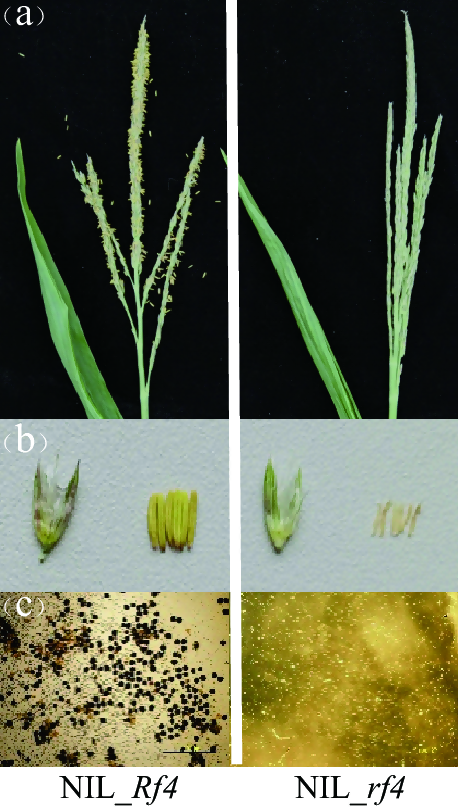

Supplement: Supplementary file 2 — Comparisons of male-fertile tassels (a), anthers (b), and pollen (c) between NIL_Rf4 and NIL_rf4. In Fig. (c), the pollen grains were stained with 1% (w/v) KI-I2. (TIF 787 kb) [file 12870_2018_1409_MOESM2_ESM.tif]

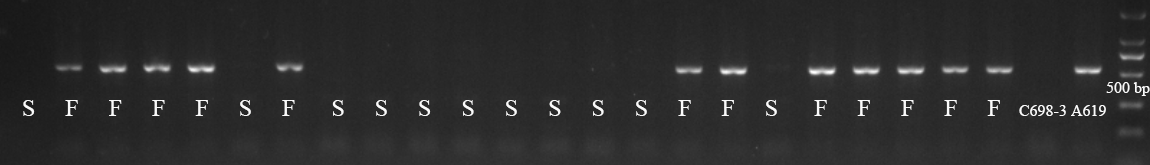

Supplement: Supplementary file 4 — Genotype analysis of the BC5 population via the dominant Rf4 tightly linked marker (5′-CGCACCTAACCGTCTCC-3′, 5′-GCGCAAGTACGCCGTAC-3′). F, fertile individuals; S, sterile individuals. (TIF 129 kb) [file 12870_2018_1409_MOESM4_ESM.tif]

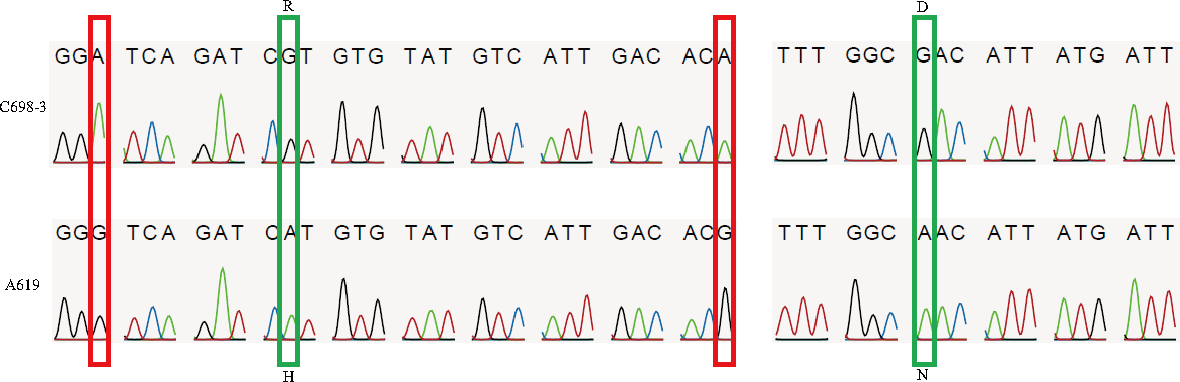

Supplement: Supplementary file 5 — Validation of SNPs within the coding sequence of GRMZM2G122850. (TIF 191 kb) [file 12870_2018_1409_MOESM5_ESM.tif]

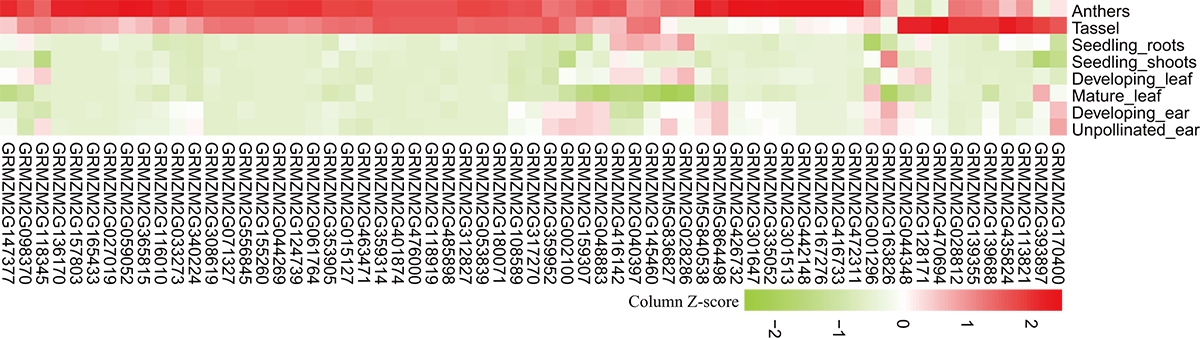

Supplement: Supplementary file 11 — Expression patterns of male reproduction-related DEGs. Their expression values (FPKM) were retrieved from the qTeller website (www.qteller.com), and the FPKM values were log2 transformed by (FPKM + 1); a Z-score was then calculated for each gene. (TIF 298 kb) [file 12870_2018_1409_MOESM11_ESM.tif]

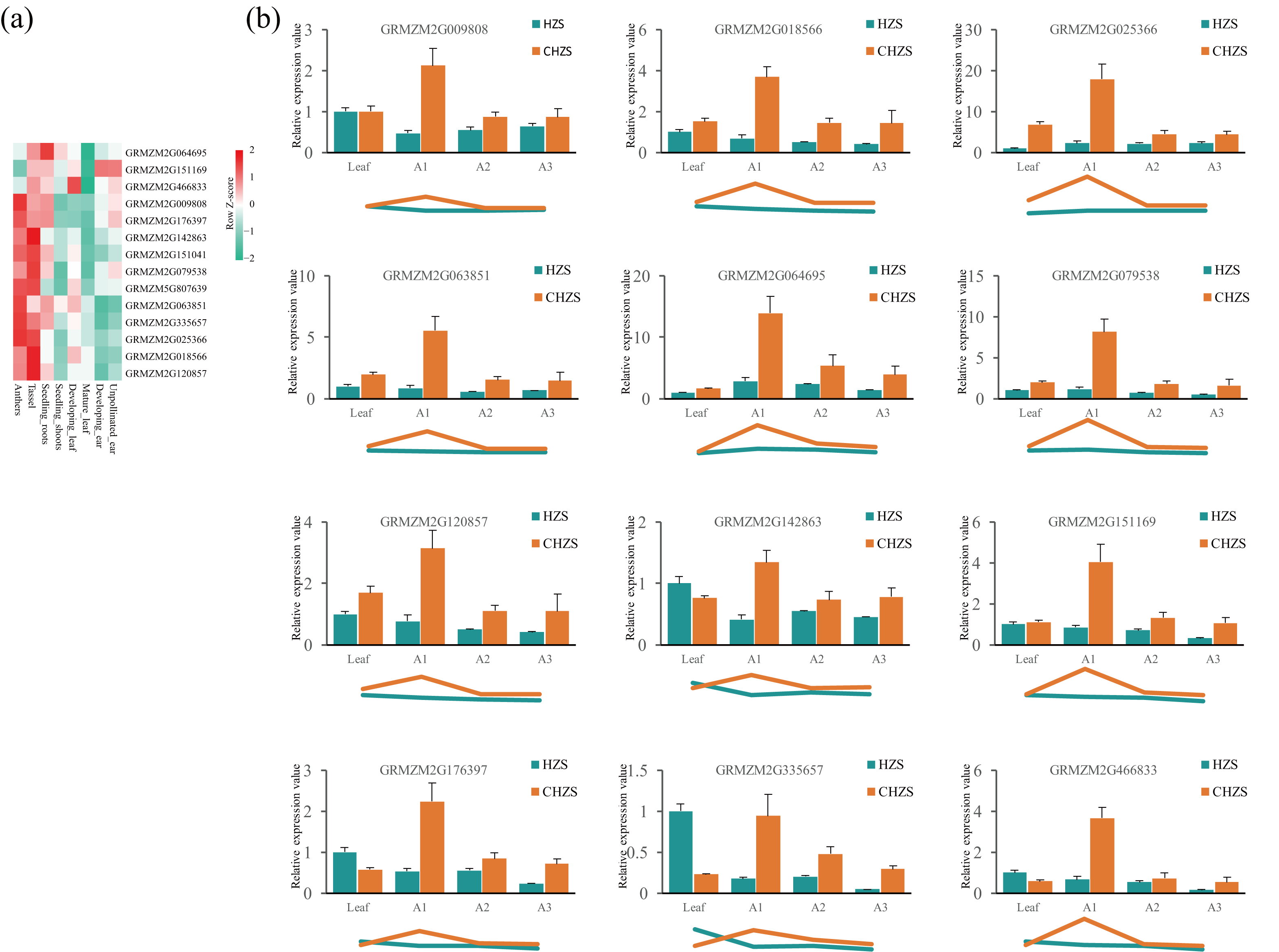

Supplement: Supplementary file 13 — (a)Tissue expression profile of 14 TCA cycle-related DEGs. The expression data (FPKM values) were obtained from qTeller (www.qteller.com). The FPKM values were log2 transformed (FPKM + 1), and a Z-score was calculated for each gene. (b) Comparison of TCA-related DEGs expression levels between the male-sterile line CHZS and its maintainer line HZS. A1, A2, and A3 denote developing anthers with the length of 1.5~ 2.0 mm (meiosis I), 2.0~ 2.5 mm (meiosis II) and 2.5~ 3.0 mm (uninucleate microspore) respectively. The data are given as means ± SEMs of at least three biological replicates. The relationship between maize pollen development stage and anther lenght can be found in another report [53]. (TIF 466 kb) [file 12870_2018_1409_MOESM13_ESM.tif]
